# Statin therapy in multimorbid older patients with polypharmacy- a cross-sectional analysis of the Swiss OPERAM trial population

**DOI:** 10.3389/fcvm.2023.1236547

**Published:** 2023-09-21

**Authors:** Luise Adam, Oliver Baretella, Martin Feller, Manuel Raphael Blum, Dimitrios David Papazoglou, Benoit Boland, Drahomir Aujesky, Stéphanie Baggio, Nicolas Rodondi

**Affiliations:** ^1^ Institute of Primary Health Care (BIHAM), University of Bern, Bern, Switzerland; ^2^ Department of General Internal Medicine, Inselspital, Bern University Hospital, University of Bern, Bern, Switzerland; ^3^ Division of Angiology, Gefässzentrum, Kantonsspital Baden, Baden, Switzerland; ^4^ Geriatric Medicine, Cliniques Universitaires Saint-Luc, Brussels, Belgium; ^5^ Health Science Research Institute, UCLouvain, Louvain, Belgium; ^6^ Population Health Laboratory (#PopHealthLab), University of Fribourg, Fribourg, Switzerland

**Keywords:** polypharmacy, multimorbidity, cardiovascular prevention, statin, -guideline adherence

## Abstract

**Background:**

Statin therapy in multimorbid older individuals with polypharmacy is controversial, particularly in primary prevention of cardiovascular disease. Thereby, physicians must weigh potential benefits against potential side effects, drug-drug interactions, and limited life expectancy.

**Aim:**

To assess the prevalence and determinants of potentially inappropriate statin therapy in multimorbid older patients.

**Methods:**

We conducted a cross-sectional analysis of patients aged ≥70 years with multimorbidity and polypharmacy in the Swiss study center of OPERAM, a cluster-randomized trial on pharmacotherapy optimization to reduce drug-related hospital admissions. We assessed potential underuse (no statin but formal indication) and potential overuse (statin but no formal indication, including predicted >60% one-year mortality based on the Walter Score) based on current guidelines for patients in secondary and primary cardiovascular prevention. We assessed the association of potential statin overuse and underuse with six patient characteristics (age, gender, number of diagnoses, number of medications, mental impairment, being housebound) in LASSO-selection analyses.

**Results:**

Of 715 multimorbid older adults (79.7 ± 6.5 years, 39.9% women), 337 (47%) were on statin. Statin therapy was appropriate in 474 (66.3%), underused in 130 (18.2%), and overused in 111 (15.5%) patients. In participants in secondary cardiovascular prevention (*n* = 437), being female (odds ratio [OR] 2.65, 95% confidence interval [CI] 1.67–4.22) was significantly associated with potential underuse while being housebound (OR 3.53, 95%CI 1.32–9.46) and taking ≥10 medications (OR 1.95,95%CI 1.05–3.67) were associated with potential overuse. In participants in primary cardiovascular prevention (*n* = 278), 28.1% were potentially under- (9%) or overusing (19%) a statin, with no identified risk factor.

**Conclusion:**

A third of hospitalized multimorbid older patients with polypharmacy potentially (either) overused or underused statin therapy. Among patients in secondary cardiovascular prevention, women were at risk for potential statin underuse. Housebound patients and those taking ≥10 medications were at risk for potential overuse of a statin. Physicians should carefully evaluate the indication for statin prescription in multimorbid older patients with polypharmacy.

## Introduction

As people age, they are increasingly likely to suffer from more than one disease (multimorbidity): 60% of adults >65 suffer from more than three chronic diseases (1). These multimorbid patients may take different drugs to treat each disease (polypharmacy) (2), some of which may be inappropriate (3). Multimorbid patients are also at risk of underusing drugs (4). Both under- and overuse (inappropriate prescribing) may lead to avoidable hospital admissions and reduce the patient's quality of life (5–7). Therefore, polypharmacy among the multimorbid older patients must be managed carefully to meet the needs of the aging population. Multimorbidity, older age, and polypharmacy are associated with cardiovascular risk factors and disease (8): more than 50% of multimorbid patients have cardiovascular disease (CVD) (9) and are often prescribed statins to reduce cardiovascular events (CVE) and mortality in primary and secondary prevention (10, 11).

Statins are effective for secondary prevention of CVD even in old patients, with data until 82 years (12), but guidelines still suggest physicians consider individual factors that could influence their decision to start, change, or end statin therapy in older patients (13, 14). A small randomized controlled trial even showed that stopping statins in patients with life-limiting disease was safe, had no significant effect on mortality, and slightly increased quality of life (15, 16).

For primary prevention of atherosclerotic CVD, the current European Society of Cardiology and American Heart Association (AHA) guidelines recommend statins to match individual cardiovascular risk, setting the minimum bar at ≥5 to <10% 10-year risk of fatal and non-fatal atherosclerotic CVD (ASCVD) (17–19). Cardiovascular risk can be calculated with tools like the PROCAM-SCORE or SCORE2 (20, 21), which include age, gender, and known modifiable cardiovascular risk factors. Among older adults, the evidence of benefits of statins for primary prevention is less clear. The recent ESC guidelines for elderly recommend risk-factor treatment in older patients (≥70 years) with very high-risk for CVD (≥15% 10-year risk of CVD according to SCORE-OP2,) and apparently healthy older high-risk patients (7.5%–15% 10-year CVD risk, Class IIa recommendation) but there is insufficient evidence for low-density lipoprotein cholesterol (LDL-C) targets (22). The AHA guidelines neither suggest nor discourage statin prescription as primary prevention for this group of patients and they encourage physicians to individualize their recommendations, taking into account life expectancy, patient preferences, co-morbidities, and other factors (evidence level E) (13, 18, 19).

Based on current evidence, physicians should carefully consider whether to prescribe drug treatment in multimorbid older patients with polypharmacy since data have shown that such patients are at risk for inappropriate prescription (underuse and overuse) (3). To date, there is little data on whether statins are appropriately prescribed to multimorbid older patients with polypharmacy, and risk factors associated with statin under- and over prescription.

To overcome this research gap, we conducted a cross-sectional analysis of the OPERAM trial (OPtimising thERapy to prevent Avoidable hospital admissions in Multimorbid older people) to assess the prevalence and determinants of potentially inappropriate statin therapy in multimorbid older hospitalized patients.

## Methods

We included participants from the Swiss study center (Bern University Hospital) of the cluster-randomized controlled OPERAM trial with detailed information on statin use, related factors and lipid values. A detailed protocol of OPERAM and its main results were published elsewhere (3, 23). We included only Swiss participants as the adjudication algorithm was based on local guidelines. Since the OPERAM trial studied a systematic drug review vs. usual care, we chose a cross-sectional study design at hospital admission, prior to the drug review, in order to receive the most accurate real life reflection.

### Study population

The OPERAM trial included multimorbid (more than three diagnosed chronic diseases) patients ≥70 years old with polypharmacy (five or more chronic medications). Patients were excluded from this analysis if they were missing information essential for assessing appropriateness of the statin therapy (e.g., missing lipid values for primary prevention).

### Outcomes: statin therapy appropriateness

Patients were classed according to ICD-10 coded diagnoses at baseline (Table 1) into patients with and without ASCVD. We classed patients with an ICD-10 code for ASCVD into secondary prevention and patients without ASCVD into primary prevention.

**Table 1:** Baseline characteristics.

	All patients (*n* = 715)	Primary prevention (*n* = 278)*	Secondary prevention (*n* = 437)**
Age [years] [means and sd]	79.7 ± 6.5	79.4 ± 6.6	79.9 ± 6.5
Sex [*n* and %]			
Men	429 (60.0)	148 (53.2)	281 (64.3)
Women	286 (40.0)	130 (46.8)	156 (35.7)
Smoking [*n* and %]	64 (9.0)	24 (8.6)	40 (9.2)
Hypertension [*n* and %]	552 (77.2)	211 (75.9)	341 (78.0)
Family history of myocardial infarction [*n* and %]	13 (8.2)	3 (5.4)	10 (9.8)
Diabetes [*n* and %]	235 (32.9)	80 (28.8)	155 (35.5)
No of medications [*n* and %]			
≥10	399 (55.8)	139 (50.0)	260 (59.5)
No. of diagnoses [*n* and %]			
3–10	101 (14.1)	60 (21.6)	41 (9.4)
≥10	614 (85.9)	218 (78.4)	396 (90.6)
Walter score >6 (scale 0–20) [*n* and %]	194 (27.1)	89 (32.0)	105 (24.0)
Housebound [*n* and %]	36 (5.0)	15 (5.4)	21 (4.81)
Dementia [*n* and %]	76 (10.6)	31 (11.5)	45 (10.3)
Statin users [*n* and %]	377 (52.7)	82 (29.5)	295 (67.5)

Sd: standard deviations.

* No diagnosis of cardiovascular disease (CVD) prior to study inclusion.

** With diagnosis of CVD prior to study inclusion.

Clinical ASCVD were defined as acute coronary syndromes, history of myocardial infarction, stable or unstable angina, coronary or other arterial revascularization, stroke, TIA, or peripheral arterial disease presumed to be of atherosclerotic origin, which occurred before study inclusion (24), and visceral atherosclerotic manifestations (Supplementary Table S2).

Patients who took a statin in accordance to current guidelines were classed as receiving appropriate statin therapy. Patients who took a statin without formal indication (overuse) or who did not receive statin therapy when statins were formally indicated (underuse) were classed as receiving “inappropriate statin therapy”.

To assess appropriateness for patients in primary prevention, we calculated the PROCAM-Score, adapted for Switzerland (25), as it is the most used risk score in Switzerland (26). We collected lipid profiles either during index hospitalization (hospitalization at the time of inclusion in the OPERAM trial) or from the patient's general practitioner (GP) whenever possible, preferably collected before lipid-lowering therapy initiation. If those values were unavailable, we used the last available lipid profile, either from their GP or from the OPERAM baseline visit. For patients with available lipid-values that were taken during statin therapy for primary prevention, we estimated the possible LDL-C value prior to statin initiation using the mean reduction values by the statin taken (27) in order to be able to better estimate the cardiovascular risk.

## Predictors

**Figure 1:**
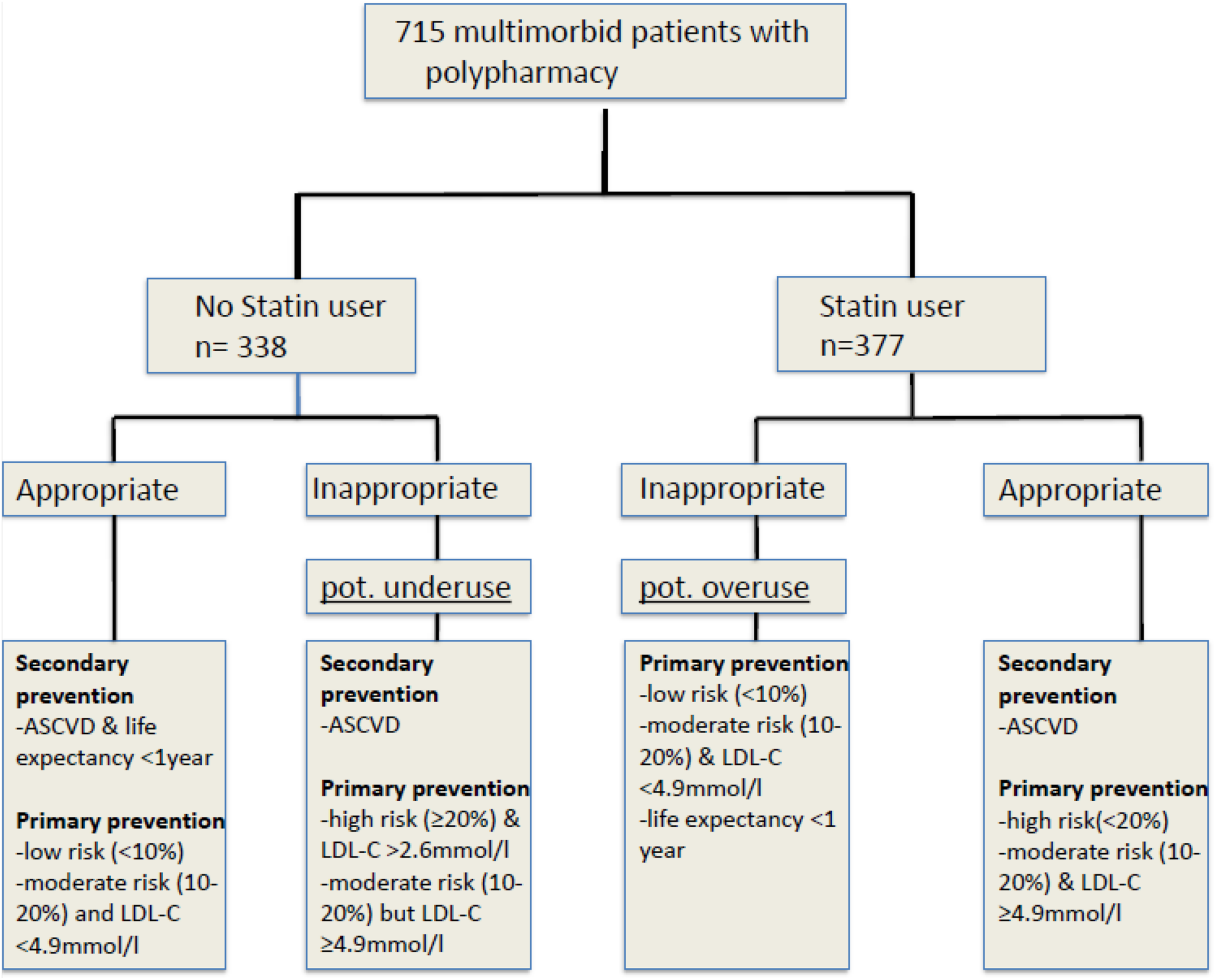
Adjudication algorithm according to AGLA score (25). Legend: abbreviations: ASCVD, LDL-C.

We considered all patients with a diagnosis of cardiovascular ischemic disease to be patients who needed secondary prevention for cardiovascular events. Thus, all patients with cardiovascular disease were considered to be underusing a statin if they were not taking a statin. For all patients, we calculated the Walter Score to determine individual 1-year mortality risk (28); we considered a score >6 (>60% 1-year mortality) to be potentially life limiting. Since current evidence suggests that statin therapy offers no benefit when prescribed for primary prevention to patients with a life-limiting disease, we classed all patients whose Walter Score was >6 as potentially being overusing statins if they were using statins at baseline (Figure 1) (15, 29).

We calculated individual risk for cardiovascular events within the next 10 years based on the patient's AGLA Score (PROCAM Score adapted for Switzerland) (25). Because several guidelines make no clear recommendation for primary prevention in patients aged >75 years (18, 19, 22, 30), we calculated cardiovascular risk scores for those patients as if they were 75 years old. Additionally, the Walter Score was used to estimate individual life expectancy (above) and two medical doctors (LA, OB) independently reviewed the patients' chart to determine if they were on appropriate statin therapy. If the two reviewers disagreed, they consulted a third reviewer (MF). Figure 1 shows the study flow chart for adjudication, which we developed based on current guidelines.

The following predictors were included into our analysis: age (classed into three categories: 70–75, 76–85 and >85 years), sex, being housebound [defined as inability to leave the house unassisted e.g., for (primary) care visits], ≥5–9 chronic medications vs. ≥10 chronic medicationsco-morbidities (into ≥10 and <10 chronic diagnoses), and whether they suffered from dementia/cognitive impairment according to ICD-10 code. All these factors potentially influence the likelihood medication will be prescribed inappropriately (31).

### Statistical analyses

We stratified patients according to their indication for statin use, i.e., primary and secondary cardiovascular prevention.

We first calculated descriptive statistics for all variables (percentages and *n* for categorical variables; means and standard deviations for continuous variables). We also calculated descriptive statistics for participants we included and excluded. Comparisons between included and excluded participants were performed using *χ*^2^ tests for categorical variables and t-tests for continuous variables.

Second, we assessed the bivariate associations between potential over- and underuse of a statin with risk factors (i.e., age sex, houseboundedness, co-morbidities and cognitive impairment) using logistic regression models, with potential underuse and overuse as two separate outcomes. We reported odd ratios (OR) with 95% confidence intervals (CI).

Third, we selected the variables with the strongest association with potential statin underuse and overuse using least absolute shrinkage and selection operator (LASSO) regression analyses. We chose this method because of the small numbers of patients in some subgroups. We used multiple logistic regression models with LASSO selection, with underuse and overuse as two separate outcomes. We also reported ORs with 95% CIs.

We used Stata Version 16.0 (StataCorp. 2019. *Stata Statistical Software: Release 16*. College Station, TX: StataCorp LLC.).

## Results

The Swiss OPERAM study center included 822 hospitalized multimorbid patients ≥70 years with polypharmacy (≥5 medications); 17 patients (2.1%) withdrew their consent; and 90 patients (10.9%) were excluded because their available information did not allow us to adjudicate the appropriateness of statin therapy. We thus included 715 patients in our analysis (see the study flow chart in the Supplementary Figure S1). Included patients had a mean age of 79.7 years [±6.5 years standard deviation (SD)]; 60% were male (Table 1). In the secondary prevention group (*n* = 437), 295 patients (67.5%) used a statin and 105 (24%) patients had a Walter score >6, 68 (23%) of those using a statin (Supplementary Table S3). In the primary prevention group (*n* = 278), 82 patients (29.5%) used statins and 89 (32%) patients had a Walter Score >6.

Additional baseline characteristics of included patients are presented in Table 1 and comparisons of the baseline characteristics of patients we included and patients we excluded because we could not properly adjudicate them in Supplementary Table S1.

In the 278 patients in primary prevention, we determined estimated risk for CVE at 10-years was low (<10%) in 95 patients, intermediate (10%–20%) in 53 patients, and high (>20%) in 40 patients (Supplementary Table S3). Patients with a Walter Score >6 (*n* = 89) were classified as potential Statin-overusers without further risk calculation.

Appropriateness of statin therapy in secondary cardiovascular prevention:

Of the 437 patients in secondary prevention, 274 (62.7%) were using a statin appropriately, 105 (24%) patients were underusing a statin, while 58 (13.3%) patients were considered to be potentially overusing a statin as their predicted mortality risk was >60% based on their Walter Score (≥6 points) (Table 2).

**Table 2:** Appropriateness of statin prescribing.

	All patients (*n* = 715)	Primary prevention (*n* = 278)	Secondary prevention (*n* = 437)
Appropriate	474 (66.3%)	200 (71.9%)	274 (62.7%)
Potential underuse*	130 (18.2%)	25 (9.0%)	105 (24.0%)
Potential overuse**	111 (15.5%)	53 (19.1%)	58 (13.3%)
LDL > 4.9 mmol/L		33 (62.3%)	

*n* and % are reported.

* Potential underuse: lack of statin therapy despite formal indication (Figure 1).

** Potential overuse: statin use without formal indication **or** if Walter Score >6 (predicted one year mortality >60%).

In secondary prevention, statin underuse was significantly associated with being very old (>85 years; OR 2.40; 95% CI 1.26–4.57) and female (OR 2.79; 95% CI1.78–4.38) in bivariate models (Table 3). We confirmed the association between underuse and being female with LASSO regression (OR 2.65; 95%CI 1.67–4.22).

**Table 3:** Associations between potential statin underuse and study variables.

	Potential underuse		Potential underuse	
	Bivariable analyses^a^		LASSO selection^b^	
	Primary prevention	Secondary prevention	Primary prevention	Secondary prevention
	p. underuse, *n* = 25	p. underuse, *n* = 105	p. underuse, *n* = 25	p. underuse, *n* = 105
Age (years)				
70–75	1 (Reference)	1 (Reference)	1 (Reference)	1 (Reference)
76–84	0.86 (0.35–2.09)	1.58 (0.89–2.80)	*	1.32 (0.74–2.40)
>85	0.41 (0.11–1.56)	***2.40*** (***1.26***–***4.57)***	*	1.86 (0.94–3.70)
Women	1.80 (0.78–4.16)	***2.79*** (***1.78***–***4.38)***	*	***2.65*** (***1.67–4.22)***
Housebound	**	0.32 (0.07–1.40)	**	0.29 (0.06–1.31)
≥10 diagnoses	0.86 (0.32–2.26)	1.94 (0.79–4.76)	*	1.81 (0.71–4.60)
≥10 medications	0.53 (0.22–1.25)	0.84 (0.54–1.30)	*	0.72 (0.45–1.16)
Dementia	0.31 (0.04–2.37)	1.49 (0.76–2.92)	*	1.23 (0.60–2.50)

LASSO: least absolute shrinkage and selection operator.

Bold values denote the significant results.

^a^ Simple logistic regressions. Odds ratios and 95% confidence intervals are reported.

^b^ Multivariable logistic regressions with LASSO selection. Odds ratios and 95% confidence intervals are reported.

* Dropped after LASSO selection.

** No housebound patients in this subgroup, removed.

Potential overuse was associated with being housebound (OR 3.58; 95% CI 1.38–9.28) and taking ≥10 medications (1.94; 95%CI 1.05–3.57) in bivariate analyses, which was confirmed after LASSO regression (OR 3.53 (1.32–9.46) and 1.95 (1.05–3.67)) (Table 4).

**Table 4:** Associations between potential statin overuse and study variables.

	Potential overuse		Potential overuse	
	Bivariable analyses^a^		LASSO selection^b^	
	Primary prevention	Secondary prevention	Primary prevention	Secondary prevention
	p. overuse, *n* = 53	p. overuse, *n* = 58	p.overuse, *n* = 53	p.overuse, *n* = 58
Age (years)				
70–75	1 (Reference)	1 (Reference)	1 (Reference)	1 (Reference)
76- 84	1.31 (0.67–2.57)	0.83 (0.43–1.59)	*	*
>85	0.55 (0.21–1.43)	1.04 (0.48–2.23***)***	*	*
Women	0.70 (0.38–1.28)	0.43 (0.22–0.83)	*	0.38 (0.19–0.76)
Housebound	1.07 (0.29–3.92)	**3.58** (**1.38–9.28)**	*	**3.53 (1.32–9.46)**
≥10 diagnoses	0.56 (0.28–1.10)	**	*	**
≥10 medications	1.39 (0.76–2.54)	***1.94*** (***1.05–3.57)***	*	**1.95 (1.05–3.67)**
Dementia	1.02 (0.40–2.63)	0.61 (0.21–1.77)	*	*

LASSO: least absolute shrinkage and selection operator.

Bold values denote the significant results.

^a^ Simple logistic regressions. Odds ratios and 95% confidence intervals are reported.

^b^ Multivariable logistic regressions with LASSO selection. Odds ratios and 95% confidence intervals are reported.

* Dropped after Lasso selection.

** No subjects in this subgroup, removed.

### Appropriateness of statin therapy in primary cardiovascular prevention

Of the 278 patients in primary prevention, 200 (71.9%) were on appropriate statin therapy when they were included in the OPERAM trial, based on our prespecified criteria (Figure 1); 25 (9%) had intermediate to high cardiovascular risk but did not take a statin so we considered them as potentially underusing a statin, while 53 patients (19.1%) were potentially overusing statin (Table 2); 23 of these potential overusers (43.4%) had a Walter Score >6.

No factor among patients in primary prevention predicted a significantly higher risk for potential under—or overuse (Tables 3, 4) in bivariate analyses. We confirmed this finding with LASSO regressions. (Tables 3, 4).

## Discussion

Among 715 multimorbid older (≥70 years) hospitalized patients, potentially inappropriate statin therapy was common (33.7%) due to potential underuse with (15.5%) and potential overuse (18.2%). Female gender was significantly associated with potential underuse in secondary prevention (OR 2.65). Being housebound (OR 3.53) and taking ≥10 medications (OR 1.95) were associated with potential overuse in secondary prevention. No specific risk factors were identified for potential over- or underuse in primary prevention.

Though the benefits of statins for primary prevention in the older patients remain controversial, statin therapy in secondary prevention in older patients is supported by evidence (22). Despite the evidence, this cross-sectional study found that 24% of patients in secondary prevention were potentially underusing statins. In secondary prevention, women, particularly, were at risk of underuse (OR 2.65), a finding that aligns with previous studies (32–34).

Statin use is clearly established for prevention of further cardiovascular events in secondary prevention, in both men and women (35). On average, women live longer than men and are therefore more likely to suffer from CVE (36). CVD is the main cause of death for women in developed countries (37, 38), but health care systems may not adequately consider women's cardiovascular risk (38). Though men and women have the same main cardiovascular risk factors, women may present differently in clinical exams, have different symptoms, and tests may not work as well for women as men (39, 40). Our findings align well with a population-based Italian study of older patients with polypharmacy, in which women were more likely to discontinue statin therapy (41).

Housebound patients in secondary prevention were at risk for overuse (defined as having a Walter Score >6) of a statin (OR 3.53), a finding that aligns with a Japanese study of eldery, housebound patient amongst whom potentially inappropriate prescribing was highly prevalent (42). This might be due to the fact that preventive medical visits are associated with a high effort by these patients and his/her proxies (42). A further factor could be that stopping an established statin therapy is currently a matter of discussion and doctors might be especially hesitant to stop a statin in a patient with established ASCVD despite advanced chronic illness and a high 1-year mortality risk (Walter Score > 6) (16). However, Kutner et al. found that stopping statins in patients with limited life expectancy was safe and slightly improved quality of life (15). In primary prevention, we found no pre-defined risk factor associated with higher risk of inappropriate statin therapy (neither potential underuse nor potential overuse), perhaps because it was more difficult to assess cardiovascular risk and define the necessity for statin therapy in the primary prevention group. Though older people are at higher cardiovascular risk, few RCTs have studied the potential benefits of statin therapy for primary prevention in the elderly. A recent meta-analysis of 28 RCTs that analyzed the benefits of statin therapy in 186,854 individuals (but only 8% aged >70 years) found that statin therapy benefited to patients over 70, but provided little evidence on patients in primary prevention (43). An age-stratified analysis of the JUPITER-trial and HOPE III study showed that taking Rosuvastatin would benefit patients >70 years regarding a composite outcome (pooled estimate HR 0.74) of nonfatal MI, stroke or cardiovascular death (44) but included only few patients >80 years. On the other hand, deprescribing statins in older patients is common in primary prevention in the very older people because clinicians might weigh the potential of side effects in frail patients higher than the potential long-term benefit (45). In an international survey using case vignettes, GPs recommended to stop statins for primary prevention especially in frail patients, those with evident side effects and patients with limited life expectancy (16). In general, evidence towards statin therapy in older people in primary prevention is limited, as shown by the discrepancies in different guidelines concerning statin therapy in the elderly (46). Especially for multimorbid older patients guidelines or recommendations concerning (deprescribing) statins are lacking (47).

Our finding of ASCVD prevalence (61%) aligns with previous studies of this older multimorbid population (8). We found statin therapy was prescribed to 52.5% in the entire patient cohort, (29.5% of patients in primary prevention and 67.5% of patients in secondary prevention). A 2011–2015 UK cohort study of statin prescription in old patients (>80 years) in primary prevention yielded similar results (30%) (45). Other studies from the UK and the US found that between 63% and 80% of the older population are taking statins (45, 32).

### Limitations

We were limited in our ability to estimate cardiovascular risk in primary prevention, so we used the PROCAM Score adapted for Switzerland, approved for maximally 75-year-old, to determine cardiovascular risk at 10 years and had two medical doctors independently judge the patient's clinical situation on a case-by-case basis (including knowledge of patient's preferences; this data was collected during the OPERAM trial) as current guidelines suggest (18). Our study was also limited because it was conducted at only one center. Though we tried to assess the statin indication for each individual patient, we did not know why treating physicians decided to prescribe or withhold a statin.

### Strengths

This is a cross-sectional analysis from the understudied, but especially in Western countries growing population of multimorbid older patients with polypharmacy. Assessing risk-factors for statin under- or overuse in this specific population of multimorbid eldery has not yet been done to our knowledge (48). We accounted for patient's specific risk factors as well as patients' individual life expectancy in a standardized way. Statins are among the most prescribed drugs for the prevention of first or recurrent cardiovascular events (49), which are highly prevalent in multimorbid older patients. This study provides evidence for risk factors that are associated with under- or overuse of a statin, alerting the clinician to frequently adapt and reassess statin therapy according to her/his patient's current situation and need.

## Conclusion

A third of multimorbid older patients with polypharmacy either potentially overused or underused statins. Among participants in secondary cardiovascular prevention, women were at highest risk for potential underuse while housebound patients were at risk for potential overuse of a statin. Physicians should carefully evaluate their need for statin prescriptions in this understudied population and adapt prescriptions as needed.

## Data Availability

The raw data supporting the conclusions of this article will be made available by the authors, without undue reservation.
