# Supplementary material for: Statin therapy in multimorbid older patients with polypharmacy- a cross-sectional analysis of the Swiss OPERAM trial population
Source: Front Cardiovasc Med. 2023 Sep 21;10:1236547. doi: 10.3389/fcvm.2023.1236547 (PMC10551156; doi:10.3389/fcvm.2023.1236547)
Supplement: Supplementary file 1 [file Datasheet1.docx]

**Supplement:**

**sFigure 1: Study Flow Chart**

Included at Swiss OPERAM study center in Bern

(n=822)

withdrawn (n=17)

included in adjudication process

(n=805)

Adjudication impossible (no lipid baseline values available)

(n=90)

included in analysis

(n=715)

**sTable 1:**

**Baseline characteristics of the excluded patients**

|  | **excluded patients (n=90)** | **included patients (n=715)** | **p-value**** |
| --- | --- | --- | --- |
| Age [years] [means and sd] | 79.3 ± 6.2 | 79.7 ± 6.5 | 0.52 |
| Sex [%]  men  women | 38 (42.2)  52 (57.8) | 429 (60.0)  286 (40.0) | 0.001 |
| Smoking [n and %] | 5 (5.7) | 64 (9.0) | 0.30 |
| Hypertension [%] | 61 (67.8) | 552 (77.2) | 0.05 |
| Family History of myocardial infarction [n and %] | *no information | 13 (8.2) | n.d. |
| Diabetes [n and %] | 17 (18.9) | 235 (32.9) | 0.007 |
| No of Medications [n and %] |  |  |  |
| ≥ 10 Medications [n and %] | 49 (54.4) | 399 (55.8) |  |
| ≥ 10 Diagnoses [n and %] | 65 (72.2) | 342 (47.8) | 0.003 |
| Walter Score >6 [n and %] | 6 (6.7) | 194 (27.1) | <0.001 |
| Dementia [n and %] | 14 (15.6) | 76 (10.6) | 0.16 |
| Being housebound [n and %] | 7 (7.8) | 36 (5.0) | 0.28 |
| Statin users [n and %] | 18 (20.0) | 377 (52.7) | <0.045 |
| Primary prevention [n and %] | 90 (100)* | 278 (38.9) | <0.001 |
| Secondary prevention [n and %] | 0* | 437 (68.1) | <0.001 |

Sd: standard deviations.

* Missing baseline lipid values led to study exclusion as no adjudication in primary prevention possible otherwise.

** student’s t-test for continuous variables and chi2-test for categorical variables

**sTable 2: ICD-10 Codes for definition of cardiovascular disease**

| **Type of atherosclerotic disease** | **ICD-10 Code** | **No of patients in secondary prevention (n=437)** |
| --- | --- | --- |
| Coronary artery disease | I20, I21, I22, I23, I24, I23, I24, I25, Z95.1, Z95.5 | 285 (65.4%) |
| Peripheral artery disease | I70.2 | 101 (23.2%) |
| Cerebrovascular infarction | I63, I63.1, I63.2, I63.3, I63.5 I63.6 I63.8 I63.9 | 135 (31.0%) |
| Abdominal Atherosclerosis | *I70.0, I70.01, I70.1, I70.9 * K55, K55.0, K55.1, K55.9 | 46 (10.6%) |
| Transient ischemic attack | G45, G45.0- G45.9 | 57 (13.1%) |

**sTable 3: Statin use in primary and secondary cardiovascular prevention**

|  | Primary Prevention |  |  | | | Secondary Prevention |  |
| --- | --- | --- | --- | --- | --- | --- | --- |
|  | All patients  n=278 | <10% risk*  n= 95 | 10-20% risk*  n= 53 | >20% risk*  n=40 | Walter > 6  n=90 | All patients  n=437 | Walter >6  n=105 |
| Statin users | 82 (29.5) | 23 (24.1) | 16 (30.2) | 20 (50) | 23 (25.6) | 295 (67.5) | 68 (64.8) |
| Atorvastatin | 49 (59.8) | 12 (12.6) | 9 (17.0) | 12 (30.0) | 16 (17.8) | 156 (52.9) | 39 (37.2) |
| Pravastatin | 6 (7.3) | 3 (3.2) | 1 (1.9) | 0 | 2 (2.2) | 18 (6.1) | 5 (4.8) |
| Simvastatin | 15 (18.3) | 7 (7.37) | 5 (9.4) | 3 (7.5) | 0 | 37 (12.5) | 6 (5.7) |
| Fluvastatin | 2 (2.44) | 0 | 0 | 2 (5.0) | 0 | 5 (1.7) | 1 (1) |
| Rosuvastatin | 10 (12.2) | 1 (1.05) | 1 (1.9) | 3 (7.5) | 5 (5.6) | 79 (26.8) | 17 (16.2) |

N and % are reported.

*10-year risk for cardiovascular events (CVE) according to PROCAM (1) adapted for Switzerland (AGLA-Score), <https://www.agla.ch/de/rechner-und-tools/agla-risikorechner>

(20) (25)
